# The effects of resistance training on bone mineral density and bone quality in type 2 diabetic rats

**DOI:** 10.14814/phy2.14046

**Published:** 2019-03-27

**Authors:** Aoi Ikedo, Kohei Kido, Satoru Ato, Koji Sato, Ji‐Won Lee, Satoshi Fujita, Yuuki Imai

**Affiliations:** ^1^ Faculty of Sport and Health Science Ritsumeikan University Shiga Japan; ^2^ Division of Integrative Pathophysiology Proteo‐Science Center Ehime University Ehime Japan; ^3^ Section of Molecular Physiology Department of Nutrition, Exercise and Sports Faculty of Science University of Copenhagen Copenhagen Denmark; ^4^ Laboratory of Sports and Exercise Medicine Graduate School of Human and Environmental Studies Kyoto University Kyoto Japan; ^5^ Department of Life Science and Applied Chemistry Nagoya Institute of Technology Nagoya Japan; ^6^ Graduate School of Human Development and Environment Kobe University Kobe Japan; ^7^ Division of Bio‐Imaging Proteo‐Science Center Ehime University Ehime Japan; ^8^ Department of Pathophysiology Graduate School of Medicine Ehime University Ehime Japan; ^9^ Division of Laboratory Animal Research Advanced Research Support Center Ehime University Ehime Japan

**Keywords:** Bone mass, bone quality, resistance training, type‐2 diabetes mellitus

## Abstract

Resistance training (RT) has been known to be effective in maintaining and improving bone strength, which is based on bone mineral density (BMD) and bone quality. However, it is not clear whether RT is effective in improving bone strength in patients with type‐2 diabetes mellitus (T2DM), who have a high risk of fracture. Therefore, we tested the effects of a 6‐week RT regimen using percutaneous electrical stimulation in T2DM model rats, male Otsuka Long‐Evans Tokushima Fatty (OLETF), and its control, Long‐Evans Tokushima Otsuka (LETO). After 6 weeks of RT, tibial BMD in RT legs was significantly higher than that in control (CON) legs in both groups. In diaphyseal cortical bone, bone area/tissue area, and cortical thickness was significantly increased in RT legs compared with CON legs in both groups. Cortical porosity was highly observed in OLETF compared with LETO, but RT improved cortical porosity in both groups. Interestingly, trabecular number, trabecular thickness and trabecular space as well as BMD and bone volume/tissue volume in proximal tibial metaphyseal trabecular bone were significantly improved in RT legs compared with CON legs in both groups. In contrast, connectivity density and structural model index were not affected by RT. These results indicate that the 6‐week RT regimen effectively increased BMD and improved bone quality in T2DM model rats as well as control rats. Therefore, RT may have the potential to improve bone strength and reduce fracture risk, even in patients with T2DM.

## Introduction

Osteoporosis is a skeletal disorder characterized by compromised bone strength and is predisposing to an increased risk of fracture (NIH Consensus Development Panel 2001). Bone strength is defined by bone mineral density (BMD) and bone quality. Bone tissues are maintained by bone remodeling with bone resorptive osteoclasts, bone‐forming osteoblasts, and matrix‐buried osteocytes. However, the dysregulation of these cellular functions induced by various genetic and/or environmental factors or systemic diseases may affect bone strength.

The number of patients with type‐2 diabetes mellitus (T2DM) has been increasing and the risk of fragility fractures is high in patients with T2DM. A previous meta‐analysis and systematic review determined that the risk of hip fracture in patients with T2DM is higher than that in non‐T2DM controls, even though their bone mineral density (BMD) is normal or higher (Janghorbani et al. 2007; Vestergaard 2007).

The increased risk of fracture in T2DM patients was attributed to the deterioration of bone quality, including impaired bone structure, material properties, and bone remodeling, which are not detected in BMD measurements (Farr et al. 2014; Farr and Khosla 2016). It has been reported that T2DM appears to negatively affect the cortical structure of bone. Increased cortical porosity is the most consistently reported alteration in the cortical bone structure in patients with T2DM (Burghardt et al. 2010; Farr et al. 2014; Yu et al. 2015), particularly in those who have experienced a fracture (Patsch et al. 2013). Furthermore, the trabecular bone scores (TBS), which are related to the bone microarchitecture and have been recently used for fracture prediction independent of BMD, are lower in patients with T2DM (Dhaliwal et al. 2014; Kim et al. 2015). A bone histomorphometric study reported that osteoblastic bone formation was decreased in the intracortical, endocortical, and cancellous surfaces of bone biopsies of patients with T2DM (Manavalan et al. 2012). Also, high glucose levels inhibited the differentiation and function of osteoblastic cell line MC3T3‐E1 (Cunha et al. 2014). Furthermore, it has been reported that the morphology and function of osteocytes, which are known to act as organizers in bone metabolism, were impaired by T2DM. A study on high fat‐fed diabetic mice revealed that the volume of osteocytes was increased and the network topology was dysregulated in diabetic mice (Mabilleau et al. 2016). Wnt/*β*‐catenin signaling stimulates bone formation, and sclerostin is produced by osteocytes and works as an antagonist for Wnt ligands. Elevated expression of the gene encoding sclerostin (*SOST*) was observed in osteocyte‐like MLO‐Y4 cells under high glucose condition, and increased concentration of serum sclerostin was observed in T2DM rats (Kim et al. 2013). For these reasons, T2DM has the potential to lead to bone fragility.

In contrast, resistance training (RT) promoted bone formation and improved BMD and bone structure in people and animals (Nickols‐Richardson et al. 2007; Swift et al. 2010; Tan et al. 2014). Mechanical loading stimulated the Wnt/*β*‐catenin signaling pathway and accelerated bone formation by reducing sclerostin expression (Moustafa et al. 2012; Tu et al. 2012). Among various exercise methods, resistance training could achieve stronger load on bone (Kohrt et al. 2004) than other representative methods, and it has been reported that resistance training using electrical stimulation could improve intramuscular glucose metabolism (Kido et al. 2016, 2018). Therefore, we hypothesized that resistance training has beneficial effects on bone quality, which is reduced by T2DM. However, the effect of RT on bone structure and quality in T2DM remains unclear.

Therefore, this study aimed to investigate the effects of resistance training using electrical stimulation on BMD, and bone structure and osteocyte morphology which indicates bone quality, in a T2DM rat model.

## Materials and Methods

### Animals and animal treatments

The study protocol was approved by the Ethics Committee for Animal Experiments at the Ritsumeikan University (Shiga, Japan). Male Long‐Evans Tokushima Otsuka (LETO) rats and Otsuka Long‐Evans Tokushima Fatty (OLETF) rats (4 weeks of age) were obtained from Japan SLC (Shizuoka, Japan). OLETF and LETO rats were used as a T2DM model and control, respectively (Kawano et al. 1992). Animals were maintained at 22–24°C with 12‐h light/dark cycles. Food (CE‐2; CLEA Japan, Tokyo, Japan) and water were available ad libitum. LETO (*n *=* *5) and OLETF (*n *=* *7) rats (20 weeks of age) completed 18 sessions of resistance exercise (RE) over a period of 6 weeks. At least 72 h after the final session, overnight‐fasted animals were euthanized, and the blood, tibial bones, and skeletal muscles were then sampled. Bone analyses were performed for the rats, in which RT was successfully performed evaluating by muscle hypertrophy.

### Resistance training (RT) protocol

RT was monitored as previously described (Ogasawara et al. 2013; Kido et al. 2018). Briefly, under isoflurane anesthesia, the whole right gastrocnemius muscle (RT leg) was subjected to maximal isometric contraction by percutaneous electrical stimulation (5 sets of 3‐sec stimulations × 10 contractions/set with a 7‐sec interval between contractions and a 3‐min rest between sets) with an electric stimulator and isolator (SS‐104J; Nihon Kohden, Tokyo, Japan). The voltage (~30 V) and stimulation frequency (100 Hz) were adjusted to produce maximal isometric tension. The left gastrocnemius muscle (CON leg) was not stimulated and served as a nonexercise control. RT performed three times a week for 6 weeks (total 18 sessions). Seventy‐two hours after the last exercise session (18 session), rats were euthanized by over anesthesia. Target tissues were removed immediately after death. Tissues were rapidly frozen in liquid nitrogen and stored at −80°C until analysis. Training effect was compared between right leg (training leg) and left leg (control leg) in the same individual. In a previous study, 18 sessions of resistance exercise caused approximately 10% skeletal muscle hypertrophy (Ogasawara et al. 2013). Additionally, our resistance exercise model induced intramuscular signaling activation and muscle protein synthesis that are similar to those observed in humans (Phillips et al. 1997; Dreyer et al. 2006; Ogasawara et al. 2013; Kido et al. 2016). Thus, we assumed that this rat resistance exercise model was suitable for evaluating the resistance exercise response in humans.

### Intraperitoneal glucose tolerance test (IPGTT)

IPGTT was performed as described previously (Kido et al. 2018). Briefly, after overnight fasting and under isoflurane anesthesia, glucose (2 g/kg) was injected intraperitoneally, and whole blood glucose was monitored for 2 h. Blood samples were collected from the tail vein, and glucose levels were measured with a glucose meter (Breeze 2; Bayer, Leverkusen, Germany).

### Radiological examination

The areal bone mineral density (aBMD) of isolated tibiae was measured by dual‐energy X‐ray absorptiometry (DXA) using a bone mineral analyzer (DCS‐600EX, ALOKA). Micro Computed Tomography scanning of the tibiae was performed according to the manufacturer's instructions using a Scanco Medical *μ*CT35 System (SCANCO Medical) with an isotropic voxel size of 10 *μ*m for cortical and trabecular analyses. For cortical bone, the ROI was set at 11.6 mm around the middle part of tibial diaphysis, and the tibial diaphysis was scanned and analyzed in a 10 *μ*m resolution (Fig. 2A). For trabecular bone, two hundred slices of proximal tibial metaphysis starting at the distal end of the growth plate were scanned and analyzed (Fig. 5A). Three‐dimensional reconstructions were generated and analyzed according to the manufacturer's instructions and the guidelines (Bouxsein et al. 2010). Micro‐CT was used to determine cortical structure (bone area (BA), tissue area (TA), bone volume (BA/TA), volumetric bone mineral density (vBMD), cortical thickness (Ct.Th), second moment of area around the major (*I*_max_) and minor (*I*_min_) axis, polar moment of inertia (*J* = *I*_max_ + *I*_min_)) and trabecular structure (bone volume (BV/TV), bone mineral density (BMD), trabecular number (Tb.N), trabecular thickness (Tb.Th), trabecular separation (Tb.Sp), connective density (Conn.D), and the structural model index (SMI)) according to the guidelines (Bouxsein et al. 2010). Cortical porosity was analyzed from 580‐slice image of cortical bone *μ*CT image using image J software. Cross sectional images of *μ*CT were first converted to binary image, followed by segmentation into bone and pores using a fixed threshold. The margin of cortical area was then traced and its area determined. Results were expressed as the summed area of all pores per cortical bone area (Po.Ar/Ct.Ar, %).

### Histological examination

Specimens of the rat tibial tissues were fixed in 70% ethanol and decalcified, then embedded in paraffin wax. Seven‐micrometer sections were cut sagittally along the tibia long axis and collected on glass slides, deparaffinized, and subjected to hematoxylin and eosin (H.E.) staining using standard protocols. After mounting with coverslips, the specimens were viewed and analyzed under a light microscope. The number of osteocytes was measured using an image analysis system (OsteoMeasure, OsteoMetrics, Inc.). Osteocyte number (N.Ot) was measured in three series regions of 200 × 400 *μ*m area in each bone section from images captured with a 20× magnification objective, as depicted in Figure 4A. Results were expressed as numbers per area (N.Ot/mm^2^).

### Immunohistochemistry

To determine the morphology of the osteocytes in the cortical bone, the same sections were deparaffinized using xylene and rehydrated using a graded series of alcohol concentrations. For antigen retrieval, the sections of rat tibia were treated with 0.1% trypsin digestion at room temperature for 30 min for immunostaining of DMP1 and then blocked with 1% bovine serum albumin at room temperature for 60 min. The primary antibody, rabbit anti‐DMP1 (AG1J002, Takara Bio Inc.), was diluted 1:100 and incubated at 4°C overnight. Alexa Fluor 488 conjugated secondary antibody was used for immunofluorescence. Images of the specimens were acquired using the NIS‐Elements software (Nikon, Tokyo, Japan). Osteocyte aspect ratio and area were measured in endosteum side in each bone section from images captured with a 20× ‐magnification objective, as depicted in Figure 4A. Aspect ratio (ellipticity) was calculated using the longest and the shortest lengths of each osteocyte (Fig. 4E). Results were calculated from the average of 10 cells of each sample, respectively (Fig. 4G and H).

### Statistical analysis

Sample size was determined according to previous study (Ato et al. 2016; Kido et al. 2018). Analysis was performed between four and seven samples, because of technical variations of sample preparations. All results are expressed as the means ± standard errors of the means (SEMs). Unpaired Student's *t*‐tests were used to compare two groups. BMD, *μ*CT parameters, and osteocyte parameters were compared by two‐way ANOVA [groups (LETO vs. OLETF) × training (left leg vs. right leg)]. Statistical significance was achieved when the *P* value was less than 0.05.

## Results

### Animal characteristics after RT

**Figure 1:**
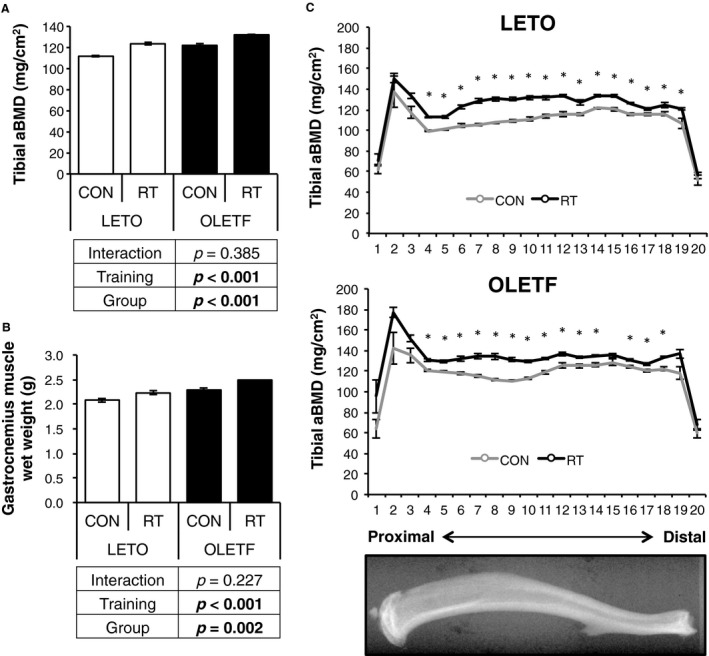
Changes in muscle wet weight and BMD by RT. (A) comparison of change in tibia BMD between OLETF and LETO. (B) comparison of change in gastrocnemius muscle wet weight between OLETF and LETO. (C) BMD of each of 20 equal longitudinal divisions of tibia from LETO and OLETF rats. Data are represented as mean ± SEM. Parameters were compared by two‐way ANOVA.

OLETF rats showed significantly higher weight and blood glucose compared with LETO rats (Table 1). Following the final training session of week 6, tibial BMD and gastrocnemius wet weight of training legs were significantly higher than control leg in both groups. In addition, OLETF was significantly higher in BMD and muscle wet weight compared with LETO, regardless of the training (Fig. 1A and B). Furthermore, when the measured tibial BMD was divided into 20 regions proximal to distal, especially the region of the diaphysis showed significantly increased BMD in RT legs compared with CON legs in both groups (Fig. 1C). Thus, the diaphyseal regions were used to analyze microarchitecture by *μ*CT. These results indicated that 6 weeks of RT was sufficient to increase muscle weight and BMDs in both LETO and OLETF rats.

**Table 1:** Animal characteristics

	LETO	OLETF
Weight (g)	393.3 ± 14.1	534.0 ± 7.5 **
Fasting blood glucose (mg/dL)	119.8 ± 3.3	154.5 ± 8.8*
Blood glucose during IPGTT (mg/dL)		
60 min	231.3 ± 12.8	302.5 ± 11.0**
120 min	106.5 ± 2.6	201.3 ± 28.0*

Values are represented as mean ± SEM. Unpaired *t*‐test, *P < 0.05, **P < 0.01 versus LETO.

### Cortical bone structure

**Figure 2:**
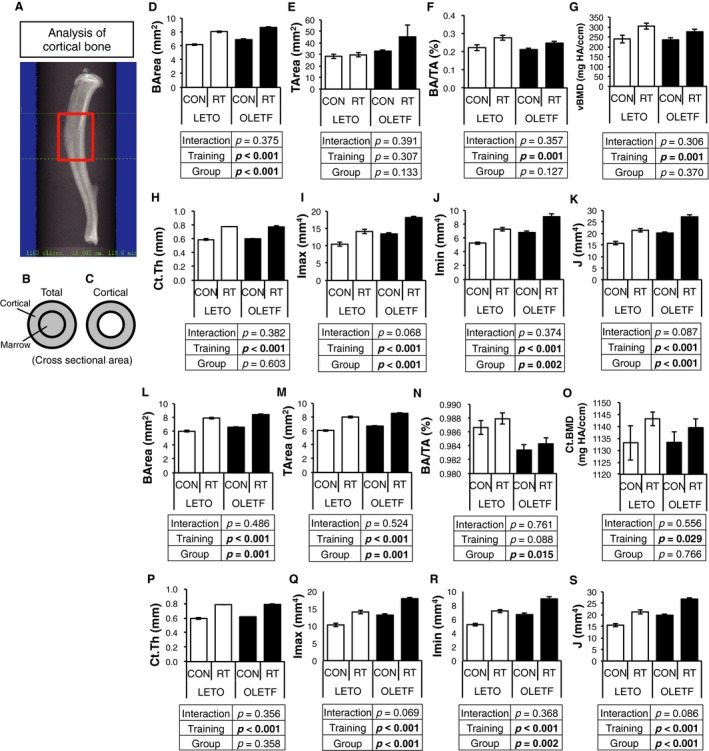
Changes in cortical bone structure of diaphysis by RT. (A) analysis region of cortical bone by *μ* CT. (D–K) bone volume (BV/TV), bone mineral density (BMD), cortical thickness (Ct.Th), second moment of area around the major (*I*_max_) and minor (*I*_min_) axis, polar moment of inertia (*J* = *I*_max_ + *I*_min_), of region including cortical and medullary cavity in diaphysis. (L–S) bone volume (BV/TV), bone mineral density (BMD), cortical thickness (Ct.Th), second moment of area around the major (*I*_max_) and minor (*I*_min_) axis, polar moment of inertia (*J* = *I*_max_ + *I*_min_) of cortical region in diaphysis. Values are represented as mean ± SEM. Data are represented as mean ± SEM. Parameters were compared by two‐way ANOVA.

The diaphysis consisting mainly of cortical bone was analyzed by *μ*CT (Fig. 2A). In the total region including the cortical and marrow area (Fig. 2B and C), the bone area (BArea) of training leg was significantly higher than control leg in both groups, and BArea of OLETF was significantly higher than that of LETO, regardless of the training (Fig. 2D). While, there is no difference in the tissue area (TA) by training and groups (Fig. 2E). In addition, the bone volume (BA/TA), vBMD and cortical thickness (Ct.Th) of training leg were significantly higher than control leg in both groups (Fig. 2F–H). The maximum moment of inertia (*I*_max_), minimum moment of inertia (*I*_min_), and polar moment of inertia (*J*) of training leg were significantly higher than that of control leg in both groups. OLETF was higher in *I*_max_, *I*_min_, and *J* than LETO, regardless of the training (Fig. 2I–K).

**Figure 3:**
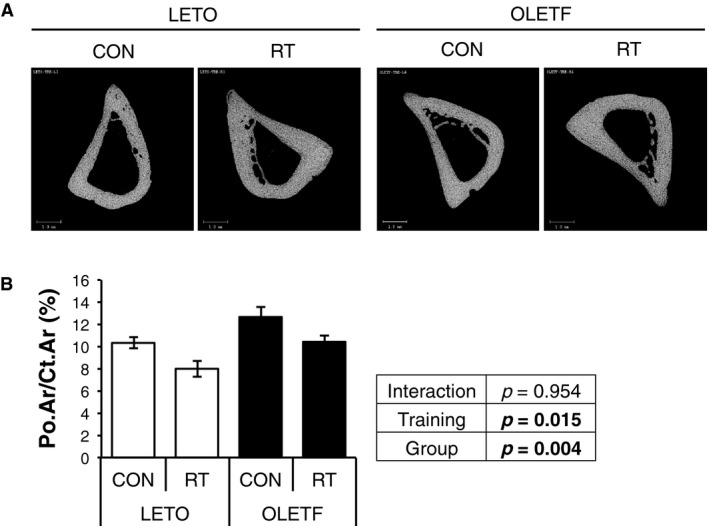
Changes in cortical porosity of diaphysis by RT. Cortical porosity was analyzed from 580‐slice image of cross sectional 2D images of cortical diaphysis. Data are represented as mean ± SEM. Parameters were compared by two‐way ANOVA.

In cortical regions (Fig. 2C), BArea and TArea of training leg were significantly higher than that of control leg in both groups. OLETF showed significantly higher BArea and TArea than LETO, regardless of the training (Fig. 2L and M). In addition, BA/TA was lower in OLETF compared with LETO (Fig. 2N). Ct.vBMD and Ct.Th of training leg were significantly higher than that control leg in both groups (Fig. 2O and P). The maximum moment of inertia (*I*_max_), minimum moment of inertia (*I*_min_), and polar moment of inertia (*J*) of training leg were significantly higher than control leg in both groups. OLETF displayed higher *I*_max_, *I*_min_, and *J* than LETO (Fig. 2Q–S). To evaluate cortical bone quality, 2D *μ*CT images were captured (Fig. 3A). Cortical porosity, which indicates bone fragility, was significantly highly observed in OLETF rats compared with LETO rats; however, RT improved in cortical porosity, regardless of the groups (Fig. 3B). These results indicated that RT could improve BMD, bone structure, and porosity of cortical bone in both groups.

### Osteocyte number and morphology in cortical bone

**Figure 4:**
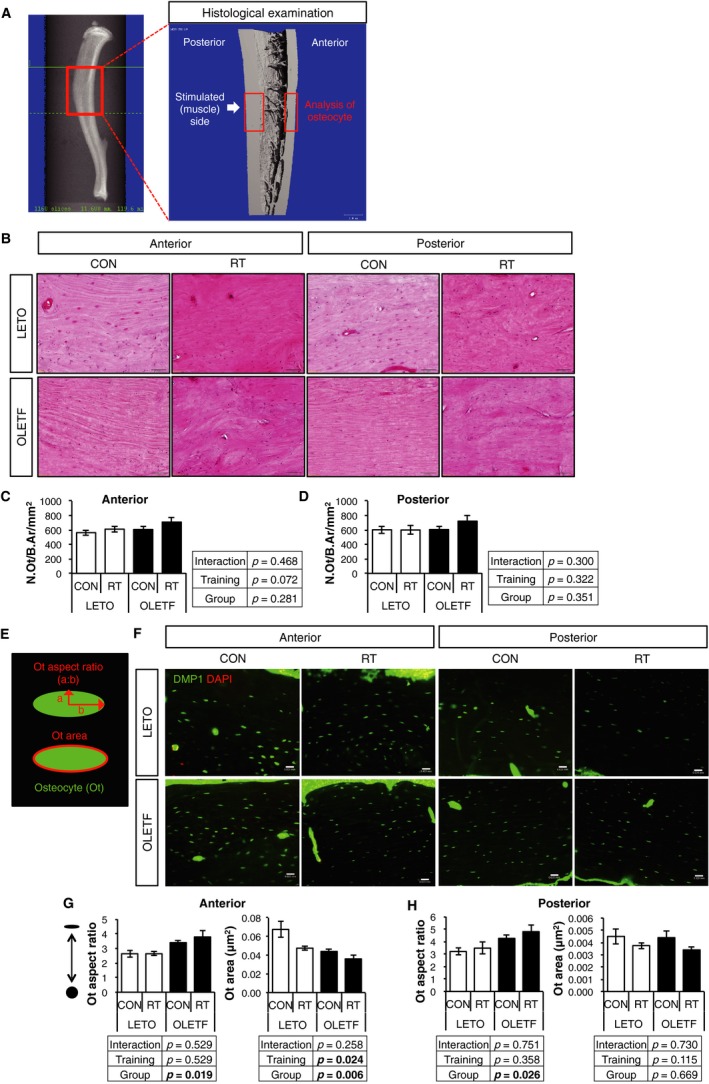
Changes in osteocyte (Ot) number and morphology by RT. (A) analysis region of cortical bone for histological examination. (B) H.E. staining of tibial diaphysis in LETO and OLETF rats. (C and D) number of osteocytes (nuclei) in (C) anterior and (D) posterior of tibial cortical bone. Scale bar, 50  *μ*m. (E) Ot aspect ratio and Ot area. (F) measurement of osteocyte morphology from tibial diaphysis in LETO and OLETF rats using immunohistochemistry against DMP1. Scale bar, 25 *μ*m. (G and H) morphology and mean area of osteocytes (DMP‐1) in (G) anterior and (H) posterior tibial cortical bone. Values are represented as mean ± SEM. Parameters were compared by two‐way ANOVA.

To histologically examine the bone quality, osteocytes in cortical bone were evaluated (Fig. 4A). Osteocyte (Ot) number and morphology were analyzed separately in anterior and posterior cortical bone. There was no difference in the number of osteocytes (N.Ot)/bone area (B.Ar) between RT and CON legs in both groups (Fig. 4B–D) regardless of bone region, anterior or posterior. On the other hand, more spindle‐shaped osteocytes were observed in OLETF compared with LETO in both sides of cortical bone (Fig. 4G and H). Osteocyte area in anterior of training leg was significantly lower than that of control leg in both groups (Fig. 4G). However, RT did not affect osteocyte area in posterior cortical bone (Fig. 4H). These results indicated that osteocytes in OLETF were more spindle‐shaped, and RT decreased osteocyte size, especially in anterior cortical bone.

### Trabecular bone structure

**Figure 5:**
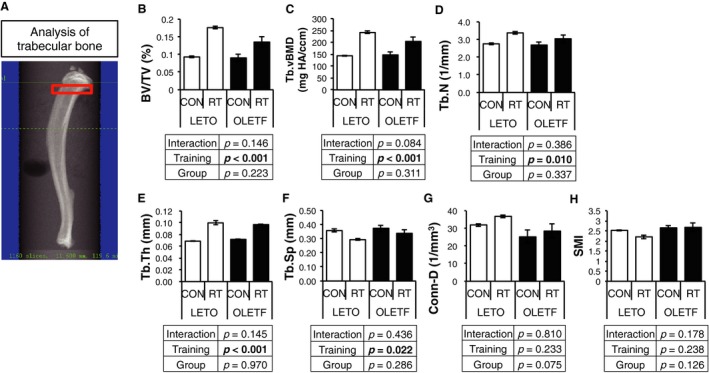
Changes in trabecular bone mass and structure in diaphysis by RT. (A) analysis region of trabecular bone by *μ* CT. (B–H) bone volume (BV/TV), bone mineral density (BMD), trabecular number (Tb.N), trabecular thickness (Tb.Th), trabecular separation (Tb.Sp), connectivity density (Conn.D), and structural model index (SMI) of trabecular bone in proximal metaphysis. Values are represented as mean ± SEM. Parameters were compared by two‐way ANOVA.

DXA analyses revealed that RT improved proximal metaphyseal BMD (Fig. 1); therefore, the trabecular bone microarchitecture in proximal tibia was analyzed by *μ*CT (Fig. 5A). Bone volume (BV/TV), Tb.vBMD, trabecular number (Tb.N), and trabecular thickness (Tb.Th) were significantly increased (Fig. 5B–E) and trabecular separation (Tb.Sp) was significantly decreased (Fig. 5F) in RT legs compared with CON legs in both groups. On the other hand, connective density (Conn‐D), and structural model index (SMI) was not changed by RT (Fig. 5G and H). These results indicated that RT could improve trabecular bone density and quality, but the effects of RT for bone quality were partially limited in OLETF and LETO.

## Discussions

This study revealed that RT improved BMD and bone structure in hyperglycemic OLETF rats, similar to LETO rats, although OLETF rats have a low cortical bone volume and a high cortical porosity.

In the present study, percutaneous electrical stimulating RT on lower legs could not affect systemic glucose metabolism. In previous study, this RT model could induce local intramuscular signal activation and muscle protein syntheses as same as resistance training for human (Phillips et al. 1997; Dreyer et al. 2006; Ogasawara et al. 2013; Kido et al. 2016). Therefore, this RT model may have the effects on bone through mechanical stress and intramuscular responses by local muscle contraction, not through systemic metabolic changes.

In a previous study, treadmill running for 12 weeks, starting just after the onset of insulin resistance (20 weeks of age), improved the cortical (Ct.Th, Ct.Ar, Ma,Ar, and Ct.Ar/Tt.Ar) and trabecular (BV/TV, Tb.N, and Tb.Sp) bone structure in OLETF rats (Ortinau et al. 2017). Our study was consistent with a previous study in spite of a short (6 weeks) training period compared with that of the previous study (12 weeks). Since the RT protocol used in this study provides direct electrical stimulation to the gastrocnemius muscle, the load intensity may be greater than while running. In addition, previous study reported that the beneficial effects of treadmill running on cortical structure was associated with an ~fourfold greater expression of *β*‐catenin protein in exercise group versus sedentary and caloric restriction group in OLETF rats (Ortinau et al. 2017), suggesting that exercise training increased Wnt/*β*‐catenin signaling in OLETF rats. Furthermore, RT used in this study has been reported to increase IGF‐1 protein expression, and improve glucose metabolism in muscle (Kido et al. 2018), which might indirectly enhance bone metabolism. From the above, we considered that RT has direct and indirect beneficial effects on improving BMD and bone structure in both cortical and trabecular bone of OLETF as well as LETO rats. In addition, it may be more beneficial training method, if systemic glucose metabolisms could be improved.

Cortical porosity, which indicates bone fragility, was higher in OLETF rats than LETO rats. These results were consistent with reports of type 2 diabetic human (Burghardt et al. 2010; Farr et al. 2014; Yu et al. 2015). In contrast, RT improved cortical porosity, independent of groups. Previous study reported that voluntary running for 9 weeks improved cortical porosity in ovariectomized and sham rats (Fonseca et al. 2011). Regardless of exercise methods, previous study was consistent with our study. Although the factors that determine cortical porosity have not been well understood, mechanical stress by RT may affect sclerostin expression in osteocyte and improve osteoblast function.

Osteocytes in OLETF showed smaller and spindle‐shaped compared with LETO in our study. In contrast, RT decreased the osteocytes area, regardless of OLETF or LETO, although RT did not change the number of osteocyte. It was reported that voluntary running for 9 weeks could increase the number of osteocytes in ovariectomized and sham rats (Fonseca et al. 2011). In this report, the authors claimed that the higher number of osteocytes was identified in the exercised animals due to not only increased osteocyte viability but also an increased pool of osteoblasts, suggesting accelerated differentiation of osteoblasts into osteocytes by mechanical stimulation. This inconsistency about alteration of osteocyte number by training between previous and our study might be caused by training duration. Six weeks in our study might not be sufficient to differentiate osteoblasts into osteocytes, because the previous studies reported that diabetic condition decreased the function of osteoblasts (Ogawa et al. 2007; Okazaki et al. 2012; Starup‐Linde et al. 2014). Furthermore, it has been reported that high fat‐fed diabetic mice showed an increased volume of osteocytes and changed network topology (Mabilleau et al. 2016); This result was also inconsistent with our study. The differences between previous studies and ours might be caused by the differences of the animal species (rats or mice), diabetic models (hyperphagia or high fat‐fed), and staining methods (DMP1 or phalloidin) for the osteocytes. To the best of our knowledge, there are only a few reports that addressed exercise‐induced osteocytic morphological changes. Therefore, the morphological changes in osteocytes induced by exercise and/or T2DM remain controversial.

In a recent GWAS meta‐analysis, the main genetic determinants of osteoporotic fracture were also reported to influence the BMD, which was the only clinical risk factor that showed a major effect on the fracture risk in the study population. These results suggest that the intervention aimed at increasing the BMD is likely to have the most clinically relevant effect on reducing the fracture risk (Trajanoska et al. 2018). From this point of view, our study suggests that RT can increase BMD and improve partial bone quality and may have a potential to effectively reduce the fracture risk in patients with T2DM.

Our study has some limitations. First, the mechanistic experiments, such as bending test, torsion test and microindentation test, were not performed in this study. These experiments are helpful to understand the effects of RT on quantitative bone strength. Second, bone remodeling, sclerostin level, advanced glycation end product (AGE) level, and oxidative stress were not evaluated. Thus, this study could not determine the reason why BMD and bone quality were improved in OLETF rats. Further analysis is required in future studies to determine whether bone quality and bone strength in T2DM can be improved by exercise.

## Conclusions

In conclusion, this study demonstrated that resistance training using percutaneous electrical stimulation increased BMD and improved bone quality in a T2DM rat model. Therefore, resistance training may be potentially effective to reduce the fracture risk in patients with T2DM.

## Conflict of Interest

No conflicts of interest, financial or otherwise, are declared by the authors.
